# Association of *SLC11A1* Polymorphisms With Tuberculosis Susceptibility in the Chinese Han Population

**DOI:** 10.3389/fgene.2022.899124

**Published:** 2022-07-22

**Authors:** Baoping Hu, Yuhe Wang, Zhongtao Wang, Xue He, Li Wang, Dongya Yuan, Yongjun He, Tianbo Jin, Shumei He

**Affiliations:** ^1^ Key Laboratory of Molecular Mechanism and Intervention Research for Plateau Diseases of Tibet Autonomous Region, School of Medicine, Xizang Minzu University, Xianyang, China; ^2^ Engineering Research Center of Tibetan Medicine Detection Technology, Ministry of Education, Xianyang, China; ^3^ Key Laboratory of High Altitude Hypoxia Environment and Life Health, School of Medicine, Xizang Minzu University, Xianyang, China; ^4^ Department of Anesthesiology, The Affiliated Hospital of Xizang Minzu University, Xianyang, China; ^5^ Department of Clinical Laboratory, The Affiliated Hospital of Xizang Minzu University, Xianyang, China; ^6^ Department of Infectious Diseases, General Hospital of Tibet Military Command, Lhasa, China

**Keywords:** tuberculosis, *SLC11A1*, polymorphism, susceptibility, case-control

## Abstract

Tuberculosis (TB) is an important health issue in the world. Although the relation of *SLC11A1* polymorphisms with TB risk has been extensively studied, it has not been reported in the northwest Chinese Han population. Therefore, this study aimed to investigate the relationships between five polymorphisms in or near the *SLC11A1* gene and susceptibility to TB. The Agena MassARRAY platform was conducted for genotyping from 510 TB patients and 508 healthy controls. Odds ratios (ORs) and 95% confidence intervals (CIs) were analyzed through logistic regression adjustment age and gender to assess the relationships between polymorphisms and TB risk. Our results identified that rs7608307 was related to increased TB risk in males (CT vs. CC: OR = 1.69, 95%CI: 1.12–2.56, *p* = 0.013; CT-TT vs. CC: OR = 1.61, 95%CI: 1.08–2.41, *p* = 0.020) and age ≤41 group (CT vs. CC: OR = 1.66, 95%CI: 1.04–2.65, *p* = 0.035), respectively. The SNP rs13062 was associated with the TB risk both in males (*p* = 0.012) and age >41 group (*p* = 0.021). In addition, we observed that the CC genotype of rs4674301 was correlated with increased TB risk in females (*p* = 0.043). Our results demonstrated the relationships between polymorphisms (rs7608307, rs4674301, and rs13062) in or near the *SLC11A1* gene and age- and sex-specific TB risk in the northwest Chinese Han population.

## Introduction

Tuberculosis (TB) is a common infectious disease with high public health impact globally. TB is predominantly caused by *Mycobacterium tuberculosis* (MTB); approximately 10% of MTB infected individuals develop clinical disease during their lifetime (Zumla et al., 2013). An estimated 1.7 billion (23%) of the world’s population is infected with MTB, leading to more than 10 million new TB cases each year (Daley, 2019). Several different factors including air pollution, smoking, drinking, poverty, undernutrition, and diabetes may affect TB development (Silva et al., 2018; Chakaya et al., 2021; Davis and Checkley, 2021). Recently, studies have demonstrated that host genetic factors also play a crucial role in the occurrence and development in TB (Ghanavi et al., 2020). Previous genome-wide association studies (GWAS) have identified multiple TB susceptibility gene loci, such as *FOXP1* (rs6786408), *AGMO* (rs916943), *ESRRB* (rs12437118), and *TGM6* (rs6114027) (Grant et al., 2016; Zheng et al., 2018). However, TB susceptibility is affected by age, sex-specific, and population-specific factors (Moller and Kinnear, 2020).

The human solute carrier family 11 member 1 (*SLC11A1*) was formerly known as natural resistance-associated macrophage protein (*NRAMP1*). *SLC11A1*, located on the human chromosome 2q35, is an important regulator of macrophage responses to MTB (McHenry et al., 2020). Genetic variants of *SLC11A1* have been reported to be significantly associated with both autoimmune and infectious disease susceptibility (Archer et al., 2015; Shahzad et al., 2022). Although numerous studies have shown that the single-nucleotide polymorphisms (SNPs) of *SLC11A1* were associated with the risk of TB in different populations (Wu et al., 2013; Harishankar et al., 2018; Asante-Poku et al., 2021), whether polymorphisms in and near the *SLC11A1* gene are associated with susceptibility to TB in the northwest Chinese Han population has not yet been reported.

Therefore, we performed this study including 510 TB patients and 508 controls to investigate the relationships between five polymorphisms (rs11695562, rs7608307, rs4674301, rs2695343, and rs13062) in or near the *SLC11A1* gene and TB risk in the northwest Chinese Han population. These results provided insights into the molecular basis for the *SLC11A1* in the occurrence and development of TB.

## Materials and Methods

### Subjects

A total of 1,018 subjects, including 510 TB patients and 508 unrelated controls, from the Xi’an Chest Hospital, Shaanxi province, were enrolled between August 2019 and September 2021. Inclusion criteria included the following: the TB patients were diagnosed, following the TB diagnosis criteria developed by the Chinese Ministry of Health; the controls were confirmed to have no history of TB and no abnormal chest X-ray; all participants were unrelated Chinese Han population. Exclusion criteria included the following: both TB patients and controls with familial hereditary diseases, chronic inflammatory, HIV infection, diabetes mellitus, other respiratory diseases, autoimmune diseases, tumors, transplants, and long-term use of hormones were excluded.

### Ethical Approval

The present study was approved by the Ethics Committee of the Xi’an Chest Hospital (No. 2012–15) and was conducted according to the principles of the Declaration of Helsinki. All the participants signed written informed consents for blood sample collection and molecular analysis.

### Sample Size Calculation

We used G*Power software to estimate the sample size of the case group and the control group by independent sample *t*-test, and the parameters were set as follows: Tail = two, Effect size = 0.20, *α* = 0.05, Power = 0.89, and Allocation ratio = 1.

### Single-Nucleotide Polymorphism Selection and Genotyping

The GoldMag DNA Purification Kit (GoldMag, China) was used to isolate the genomic DNA from samples, according to the manufacturer’s instructions. DNA purity and concentration were detected using the NanoDrop 2000 spectrophotometer (Thermo Fisher, United States). We selected the *SLC11A1* gene as a candidate gene for the study, according to previously published studies (Meilang et al., 2012; Harishankar et al., 2018; Shahzad et al., 2022) and randomly selected five unreported SNPs (rs11695562, rs7608307, rs4674301, rs2695343, and rs13062) in or near *SLC11A1* with the global minor allele frequency (MAF) greater than 0.05 from the 1000 Genomes Project data (http://www.internationalgenome.org/). The amplification and extension primers of each SNP were designed by Agena Bioscience Assay Design Suite V2.0 software. Moreover, the SNP genotype and data management were performed by the MassARRAY iPLEX platform and Agena Bioscience TYPER 4.0 software, respectively.

### Statistical Analysis

The demographic characteristics of subjects involving age and sex were performed by SPSS 20.0 software. The *t*-test was used to detect the difference between the two groups for continuous variables, and the chi-squared test was used to detect the difference of the categorical variables between the two groups. Hardy–Weinberg equilibrium (HWE) *p-*values of the control group and differences in frequencies of allele and genotype distribution between the two groups were obtained from the χ^2^ test. Logistic regression analysis was used to calculate the odds ratios (ORs) and 95% confidence interval (CI) values for estimating the association between SNPs and TB risk adjusting age and gender. The PLINK software (version 1.07) was used to evaluate the relationship between *SCL11A1* polymorphisms and TB sensibility under four genetic models. Furthermore, the stratified analyses by the mean age and gender were also performed. In this study, *p* < 0.05 in all statistical tests was thought to be statistically significant.

## Results

### Demographics

As summarized in Table 1, the basic demographic characteristics of subjects were described. The mean age of TB patients was 41.90 ± 14.83 years old including 318 (62.35%) males, 192 (37.65%) females, 233 (45.7%) age >41, and 277 (54.3%) age ≤41. For healthy controls, the mean age was 41.14 ± 18.42 years old containing 316 (62.20%) males, 192 (37.80%) females, 213 (41.9%) age >41, and 295 (58.1%) age ≤41. No difference was observed in mean age (*p* = 0.469) and gender (*p* = 0.961) distribution. Statistical analysis suggested that the age and gender between cases and controls matched.

**TABLE 1:** Basic characteristics of samples.

Variable	Case (*n* = 510)	Control (*n* = 508)	*p*
Age, year (mean ± SD)	41.90 ± 14.83	41.14 ± 18.42	0.469
≤41	277 (54.3%)	295 (58.1%)	0.227
>41	233 (45.7%)	213 (41.9%)	
Gender			0.961
Males	318 (62.35%)	316 (62.20%)	
Females	192 (37.65%)	192 (37.80%)	

SD: standard deviation. *p* < 0.05 indicates statistical significance.

### Overall Analysis

The detailed characteristics of five SNPs (rs11695562, rs7608307, rs4674301, rs2695343, and rs13062) in or near the *SLC11A1* gene are displayed in Table 2. All SNPs were in accordance with HWE (*p* > 0.05) among healthy controls. The allele and genotype frequency distribution of all SNPs were analyzed by the χ^2^ test, and the results are shown in Table 2 and 3. However, the five SNPs did not present any difference in the allele and genotype frequencies among TB patients and healthy controls (*p* > 0.05).

**TABLE 2:** Allele frequency distribution and association with the TB risk.

SNP-ID	Chr	Position	Role	Allele	MAF		HWE *p*-value	OR (95%CI)	*p*
					Case	Control			
rs11695562	2	218369982	Intergenic	C/T	0.280	0.284	0.101	0.98 (0.81–1.19)	0.839
rs7608307	2	218377795	Intergenic	T/C	0.108	0.097	0.804	1.12 (0.84–1.49)	0.439
rs4674301	2	218378139	Intergenic	C/T	0.191	0.191	0.886	1.00 (0.80–1.25)	0.989
rs2695343	2	218390700	Intron	A/G	0.353	0.347	0.096	1.02 (0.85–1.23)	0.795
rs13062	2	218395928	3′-UTR	A/C	0.292	0.289	0.101	1.01 (0.84–1.23)	0.890

Chr: chromosome; CI: confidence interval; HWE: Hardy–Weinberg equilibrium; MAF: minor allele frequency: OR: odds ratio; SNP: single-nucleotide polymorphism. *p* < 0.05 indicates statistical significance.

**TABLE 3:** Association between *SLC11A1* polymorphisms and the risk of TB.

SNP-ID	Model	Genotype	Case	Control	OR (95%CI)	*p*
rs11695562	Co-dominant	TT	267	268	1	
		TC	200	191	1.05 (0.81–1.37)	0.715
		CC	43	49	0.87 (0.56–1.36)	0.549
	Dominant	TT	267	268	1	
		TC-CC	243	240	1.01 (0.79–1.30)	0.916
	Recessive	TT-TC	467	459	1	
		CC	43	49	0.86 (0.56–1.32)	0.478
	Log-additive	--			0.98 (0.81–1.18)	0.818
rs7608307	Co-dominant	CC	403	414	1	
		CT	104	89	1.21 (0.88–1.66)	0.237
		TT	3	5	0.60 (0.14–2.54)	0.489
	Dominant	CC	403	414	1	
		CT-TT	107	94	1.18 (0.86–1.60)	0.304
	Recessive	CC-CT	507	503	1	
		TT	3	5	0.58 (0.14–2.45)	0.460
	Log-additive	-			1.13 (0.84–1.51)	0.417
rs4674301	Co-dominant	TT	339	333	1	
		TC	147	156	0.92 (0.70–1.20)	0.535
		CC	24	19	1.24 (0.67–2.30)	0.501
	Dominant	TT	339	333	1	
		TC-CC	171	175	0.95 (0.73–1.24)	0.712
	Recessive	TT-TC	486	489	1	
		CC	24	19	1.27 (0.69–2.35)	0.445
	Log-additive	--			1.00 (0.80–1.24)	0.971
rs2695343	Co-dominant	GG	216	225	1	
		GA	228	213	1.12 (0.86–1.46)	0.412
		AA	66	70	0.96 (0.66–1.42)	0.854
	Dominant	GG	216	225	1	
		GA-AA	294	283	1.08 (0.84–1.38)	0.548
	Recessive	GG-GA	444	438	1	
		AA	66	70	0.91 (0.63–1.31)	0.621
	Log-additive	-			1.02 (0.85–1.22)	0.850
rs13062	Co-dominant	CC	246	265	1	
		CA	230	192	1.28 (0.99–1.66)	0.061
		AA	34	51	0.71 (0.45–1.14)	0.155
	Dominant	CC	246	265	1	
		CA-AA	264	243	1.16 (0.91–1.49)	0.232
	Recessive	CC-CA	476	457	1	
		AA	34	51	0.64 (0.40–1.00)	0.050
	Log-additive	--	-	-	1.01 (0.83–1.22)	0.935

CI: confidence interval; OR: odds ratio; SNP: single-nucleotide polymorphism; TB: tuberculosis. *p* < 0.05 indicates statistical significance.

### Stratified Analysis

We performed the stratification analysis by the gender, as shown in Table 4. Analysis results suggested that rs7608307 was significantly related to increase the TB risk in males under the co-dominant model (CT vs. CC: OR = 1.69, 95%CI: 1.12–2.56, and *p* = 0.013), dominant model (CT-TT vs. CC: OR = 1.61, 95%CI: 1.08–2.41, and *p* = 0.020), and log-additive model (OR = 1.47, 95%CI: 1.01–2.13, and *p* = 0.043). The SNP rs13062 was also associated with increased TB risk in males under the co-dominant model (CA vs. CC: OR = 1.52, 95%CI: 1.10–2.12, and *p* = 0.012). The SNPs were observed to be associated with an increased risk of TB in females under the co-dominant model (CC vs. TT: OR = 3.82, 95%CI: 1.04–4.03, and *p* = 0.043) and the recessive model (CC vs. TT-TC: OR = 3.85, 95%CI: 1.06–4.02, and *p* = 0.041).

**TABLE 4:** Association between *SLC11A1* polymorphisms and the TB risk stratified by gender.

SNP-ID	Model	Genotype	Male		Female	
			OR (95% CI)	*P*	OR (95% CI)	*P*
rs7608307	Co-dominant	CC	1		1	
		CT	1.69 (1.12–2.56)	0.013	0.75 (0.46–1.23)	0.255
		TT	0.79 (0.17–3.56)	0.753	—	—
	Dominant	CC	1		1	
		CT-TT	1.61 (1.08–2.41)	0.020	0.73 (0.45–1.20)	0.219
	Recessive	CC-CT	1		1	
		TT	0.72 (0.16–3.24)	0.666	—	—
	Log-additive	--	1.47 (1.01–2.13)	0.043	0.72 (0.44–1.17)	0.185
rs4674301	Co-dominant	TT	1		1	
		TC	0.89 (0.63–1.26)	0.508	0.98 (0.63–1.51)	0.921
		CC	0.77 (0.36–1.63)	0.490	3.82 (1.04–4.03)	0.043
	Dominant	TT	1			
		TC-CC	0.87 (0.63–1.21)	0.414	1.11 (0.73–1.69)	0.626
	Recessive	TT-TC	1			
		CC	0.79 (0.37–1.68)	0.546	3.85 (1.06–4.02)	0.041
	Log-additive	--	0.88 (0.67–1.16)	0.371	1.24 (0.86–1.78)	0.249
rs13062	Co-dominant	CC	1		1	
		CA	1.52 (1.10–2.12)	0.012	0.98 (0.64–1.49)	0.914
		AA	0.76 (0.42–1.36)	0.352	0.64 (0.29–1.41)	0.269
	Dominant	CC	1		1	
		CA-AA	0.79 (0.37–1.68)	0.546	0.91 (0.61–1.37)	0.663
	Recessive	CC-CA	1		1	
		AA	0.63 (0.36–1.10)	0.104	0.65 (0.30–1.39)	0.267
	Log-additive	--	1.10 (0.86–1.40)	0.453	0.88 (0.64–1.20)	0.414

CI: confidence interval; OR: odds ratio; SNP: single-nucleotide polymorphism; TB: tuberculosis. *p* < 0.05 indicates statistical significance.

The results of age-stratified analysis showed that the CT genotype of rs7608307 was significantly associated with the increased TB risk compared with the CC genotype in the age ≤41 group (OR = 1.66, 95%CI: 1.04–2.65, and *p* = 0.035). However, the AA genotype of rs13062 was associated with the reduced risk of TB in the old group under the co-dominant genetic model (OR = 0.44, 95%CI: 0.20–0.98, and *p* = 0.043) and the recessive model (OR = 0.40, 95%CI: 0.18–0.87, and *p* = 0.021) (Table 5). However, there was no significant difference between the other SNPs and TB susceptibility (*p* > 0.05); all data were not shown.

**TABLE 5:** Association between *SLC11A1* polymorphisms and the TB risk stratified by age.

SNP-ID	Model	Genotype	≤41		>41	
			OR (95% CI)	*P*	OR (95% CI)	*P*
rs7608307	Co-dominant	CC	1		1	
		CT	1.66 (1.04–2.65)	0.035	0.99 (0.63–1.57)	0.972
		TT	0.34 (0.06–1.99)	0.232	—	—
	Dominant	CC	1		1	
		CT-TT	1.50 (0.95–2.36)	0.080	1.01 (0.64–1.60)	0.970
	Recessive	CC-CT	1		1	
		TT	0.32 (0.05–1.83)	0.198	—	—
	Log-additive	--	1.30 (0.86–1.97)	0.209	1.03 (0.66–1.62)	0.902
rs13062	Co-dominant	CC	1		1	
		CA	1.19 (0.82–1.72)	0.362	1.25 (0.85–1.86)	0.260
		AA	0.96 (0.51–1.80)	0.892	0.44 (0.20–0.98)	0.043
	Dominant	CC	1		1	
		CA-AA	1.14 (0.80–1.63)	0.458	1.08 (0.74–1.56)	0.703
	Recessive	CC-CA	1		1	
		AA	0.88 (0.48–1.63)	0.692	0.40 (0.18–0.87)	0.021
	Log-additive	--	1.06 (0.81–1.38)	0.694	0.90 (0.67–1.22)	0.498

CI: confidence interval; OR: odds ratio; SNP: single-nucleotide polymorphism; TB: tuberculosis. *p < 0.05 indicates statistical significance.

## Discussion

In this case-control study, we explored the relationships between five SNPs in or near the *SLC11A1* gene and susceptibility to TB in the northwestern Chinese Han population. The overall analysis results did not find differences in the allele and genotype frequency distributions of these five SNPs (rs11695562, rs7608307, rs4674301, rs2695343, and rs13062) in or near the *SLC11A1* gene between the TB patients and healthy control groups. However, the stratified analysis by age and gender showed that rs7608307, rs13062, and rs4674301 polymorphisms were associated with TB susceptibility (*p* < 0.05).

*SLC11A1* was identified as a proton cation antiporter, which localizes to lysosomes or late endosome. It has been reported that *SLC11A1* regulates macrophage activation and plays an important role in host innate immune response against infections (Correa et al., 2017). Numerous studies have demonstrated that *SLC11A1* polymorphisms were associated with the risk of TB. A study reported that rs3731865 (*SLC11A1*) was associated with TB risk in African-Americans, and two SNPs (rs3731863 and rs17221959) in *SLC11A1* were associated with TB risk in Caucasians (Velez et al., 2009). A significant association was found between the polymorphisms (Asn543Asp and rs17235409) in the 3′ untranslated region (UTR) of the *NRAMP1* gene and TB in Venezuelan (Fernandez-Mestre et al., 2015) and India population (Medapati et al., 2017). In addition, the results of three meta-analysis studies showed that *SLC11A1* polymorphisms ((D543N, 3′UTR TGTG ins/del, INT4, [GT]n), rs3731865, and rs17235416)) were significantly associated with TB risk (Li et al., 2011; Meilang et al., 2012; Archer et al., 2015). Recently, rs17235409 (*SLC11A1*) has been observed with an even stronger protective effect against Mtb infection in Ghana (Asante-Poku et al., 2021). *SLC11A1* genetic variation and low expressions have been reported to cause immune response impairment in TB patients (Shahzad et al., 2022).

Several studies about the associations between *SLC11A1* polymorphisms and TB risk have also been reported in the Chinese populations. There was a statistical association between 3′UTR polymorphism in *NRAMP1* (TGTG deletion) and the risk of TB in the Chinese Kazak population (Wu et al., 2013). The D543NG/A and 3′UTR TGTG+/del were related to TB susceptibility in the Chinese Han population (Wu et al., 2015). The rs17235409 (SLC11A1) was associated with the spinal TB risk in the southern Han Chinese population, and the NRAMP1 protein expression was increased in spinal TB patients (Li et al., 2022). In our study, we only observed that the CT and CT-TT genotypes of rs7608307 and the CA genotype of rs13062 were associated with the increased risk of TB in the younger group (age ≤41) and males, respectively, and the CC genotype of rs4674301 was correlated with the increased TB risk in females. Similar to our results, D543N in *SLC11A1* was correlated with the TB risk in age ≤65 years group and the females of the Hong Kong Chinese population (Leung et al., 2007). These findings may suggest the genetic susceptibility to TB differences by age and sex and emphasize the importance of considering heterogeneity in genetic and TB association studies. TB epidemiology is a sex-specific disease characterized by significant differences in prevalence between men and women worldwide (Hertz and Schneider, 2019). Sex differences in TB may be due to genetics, sex hormones, lifestyle, and other factors. Previous research found that the odds of Mtb infection increased significantly with age in young girls, and the increase in odds was borderline significant (Fernandes et al., 2018). Aging has significant effects on both the innate and adaptive immune systems, which may contribute to the increased risk of TB (Byng-Maddick and Noursadeghi, 2016). Our results are needed to verify in further studies with large samples.

There are some limitations to this study that cannot be ignored. First, this study found for the first time that rs7608307, rs4674301, and rs13062 were associated with age- and sex-specific susceptibility to TB in the northwestern Chinese Han population. The results of this study need to be replicated in more Chinese populations. Second, information of some risk factors is missing (e.g., smoking, alcohol consumption, living conditions, and pollution). We will consider these factors in future studies. Finally, this study is only a very preliminary study, and further experiments are needed to explore gene expressions and protein functions.

## Conclusion

Our results revealed the significant association between polymorphisms (rs7608307, rs4674301, and rs13062) in or near the *SLC11A1* gene with age- and sex-specific susceptibility to TB in the northwestern Chinese Han population. A larger sample size will be needed to confirm our results in further studies. The study provides an important direction to understand the occurrence and development mechanism of TB in Chinese population.

## Data Availability

The original contributions presented in the study are included in the article. Further inquiries can be directed to the corresponding author.
